# Influences of noise reduction on speech intelligibility, listening effort, and sound quality among adults with severe to profound hearing loss

**DOI:** 10.3389/fnins.2024.1407775

**Published:** 2024-07-23

**Authors:** Ruijuan Dong, Pengfei Liu, Xin Tian, Yuan Wang, Younuo Chen, Jing Zhang, Liu Yang, Shiyang Zhao, Jingjing Guan, Shuo Wang

**Affiliations:** ^1^Department of Otorhinolaryngology Head and Neck Surgery, Key Laboratory of Otolaryngology Head and Neck Surgery (Capital Medical University), Ministry of Education, Beijing Tongren Hospital, Capital Medical University, Beijing, China; ^2^Beijing Key Laboratory of Fundamental Research on Biomechanics in Clinical Application, School of Biomedical Engineering, Capital Medical University, Beijing, China; ^3^Beijing Institute of Otolaryngology, Otolaryngology-Head and Neck Surgery, Key Laboratory of Otolaryngology Head and Neck Surgery (Capital Medical University), Ministry of Education, Beijing Tongren Hospital, Capital Medical University, Beijing, China; ^4^Sonova Shanghai Co., Ltd., Shanghai, China; ^5^Department of Otolaryngology Head and Neck Surgery, Peking University People’s Hospital, Beijing, China

**Keywords:** severe-to-profound hearing loss, hearing aids, noise reduction, intelligibility, listening effort

## Abstract

**Introduction:**

Noise reduction (NR) algorithms have been integrated into modern digital hearing aids to reduce noise annoyance and enhance speech intelligibility. This study aimed to evaluate the influences of a novel hearing aid NR algorithm on individuals with severe-to-profound hearing loss.

**Methods:**

Twenty-five participants with severe-to-profound bilateral sensorineural hearing loss underwent three tests (speech intelligibility, listening effort, and subjective sound quality in noise) to investigate the influences of NR. All three tests were performed under three NR strength levels (Off, Moderate, and Strong) for both speech in noise program (SpiN) and speech in loud noise program (SpiLN), comprising six different hearing aid conditions.

**Results:**

NR activation significantly reduced listening effort. Subjective sound quality assessments also exhibited benefits of activated NR in terms of noise suppression, listening comfort, satisfaction, and speech clarity.

**Discussion:**

Individuals with severe-to-profound hearing loss still experienced advantages from NR technology in both listening effort measure and subjective sound quality assessments. Importantly, these benefits did not adversely affect speech intelligibility.

## Introduction

Severe-to-profound hearing loss patients (estimated at 87 million worldwide) may face challenges related to social participation, health complications, work or study limitations, and decline of overall life quality (Stevens et al., 2013). Several studies have found that adults with severe and profound hearing loss experienced higher levels of social isolation, anxiety, and depression compared to peers with better hearing (Grimby and Ringdahl, 2000; Hallam et al., 2006). Besides social support networks from friends and family, hearing healthcare plays a crucial role in the lives of hearing loss individuals, which provides support and effective communication tools to avoid poor health outcomes and social isolation (Carlsson et al., 2015).

If we adopt the definition of severe hearing loss as an average air-conduction threshold of above 60 dB HL across 0.25–4 kHz, the prevalence is approximately 2.5% of the population (Goman and Lin, 2016). Taking a more conservative approach by pushing up the cutoff value to 70 dB HL, the estimated prevalence decreases to 0.7% of the population (Turton and Smith, 2013; Carlsson et al., 2015). One study showed that out of 4,286 patients with severe-to-profound hearing loss, 1,323 (31%) used a unilateral hearing aid in either the right or left ear, 2,403 (58.5%) used bilateral hearing aids, totaling 3,726 (87%) participants using either unilateral or bilateral hearing aids (Turunen-Taheri et al., 2019).

The present study follows the guideline of WHO by defining severe hearing loss as an average air-conduction threshold of above 65 dB HL across 0.25–4 kHz (World Health Organization, 2021; Lin, 2024). Individuals with severe-to-profound hearing loss are often long-term, full-day users of hearing aids and heavily relying on them due to the extent of their hearing loss. Their amplification needs are unique: individuals in this group require a wide range of input levels to be audible, comfortable, and safe within their narrow residual hearing range (Convery and Keidser, 2011). Severe-to-profound hearing loss is one of the most challenging auditory problems, as affected individuals may struggle to communicate in daily life and may not hear or barely hear conversational-level speech without assistive hearing devices. Even with properly fitted hearing aids that can improve hearing, people with severe-to-profound hearing loss may still experience difficulties in understanding speech, especially in noisy environments (Flynn et al., 1998).

Hearing loss affects not only the audibility of sound but also the quality of sound perception (Moore, 1996). Among hearing aid users, one of the most common complaints is difficulty experienced when listening in noisy environments. To address this issue, noise reduction (NR) algorithms are integrated into modern digital hearing aids to alleviate noise annoyance and enhance speech intelligibility (Brons et al., 2013). These NR algorithms continuously analyze the input signal, estimate the signal-to-noise ratio (SNR), and selectively attenuate the gain in frequency regions dominated by noise to increase SNR. This improvement in SNR aims to facilitate better speech intelligibility in noise.

However, the efficacy of NR has yielded mixed results in studies that have utilized different variables, tools, and methodologies (Bentler et al., 2008; Brons et al., 2013, 2014, 2015; Ohlenforst et al., 2018; Wong et al., 2018). It’s worth noting that these studies primarily focused on participants with normal hearing (Brons et al., 2013), or those with mild to moderate hearing loss (Bentler et al., 2008; Brons et al., 2014, 2015; Ohlenforst et al., 2018; Wong et al., 2018). The impact of NR on individuals with severe-to-profound hearing loss remains unclear.

Many studies assessed the benefits of NR via speech intelligibility test. Results have exhibited wide variation, with some studies reporting no effect on speech intelligibility (Bentler et al., 2008; Brons et al., 2013, 2014; Desjardins and Doherty, 2014; Brons et al., 2015; Desjardins, 2016), but others showed significant improvement (Peeters et al., 2009; Wong et al., 2018). Besides, a subset of studies also raised concerns about potential adverse effects associated with NR processing on intelligibility (Nordrum et al., 2006; Bentler et al., 2008). These studies suggested that although background noise could be attenuated, there might also be unintended consequence of speech distortion. More aggressive signal processing might introduce more speech enhancement as well as more distortions (Loizou and Kim, 2011).

In summary, no consistent benefits of NR application in speech understanding are observed in literature so far. Recent studies have incorporated measures of listening effort, which appear to capture subtler NR influences that may be advantageous to listeners (Sarampalis et al., 2009; Brons et al., 2013; Desjardins and Doherty, 2014; Ohlenforst et al., 2017a,b, 2018). NR is designed to improve speech audibility and intelligibility in both quiet and noisy conditions, potentially reducing the listening effort.

Objective assessment of listening effort has involved physiological measurements, such as pupil response and electroencephalography (EEG; Neher et al., 2014; Ohlenforst et al., 2018; Fiedler et al., 2021). For instance, Wendt et al. (2017) investigated the effect of intelligibility level and NR schemes on listening effort as indicated by the peak pupil dilation (PPD) in a group of people with hearing impairment. The results indicated that processing effort and recognition performance were affected by both intelligibility level and NR scheme (NoNR vs. NR). Increased PPD was observed for SNR corresponding to the individual 50% correct (L50) compared to SNR corresponding to the individual 95% correct (L90), suggesting increased listening effort in L50. The application of an NR scheme resulted in reduced listening effort as indicated by smaller PPDs.

Subjective assessment of listening effort included self-reporting and questionnaire surveys, such as the Speech, Spatial and Qualities of Hearing Scale (SSQ; Gatehouse and Noble, 2004), categorical scales (Alhanbali et al., 2017; Ferschneider and Moulin, 2023), and behavioral tests like single-task or dual-task paradigms (Sarampalis et al., 2009; Neher et al., 2014; Degeest et al., 2021). Baer et al. (1993) suggested that the primary benefit of hearing aid NR might be a reduction in listening effort rather than a direct enhancement of speech clarity. Research has indicated that signal processing of modern hearing aids has the potential to reduce listening effort (Brons et al., 2014; Ohlenforst et al., 2017b; Wong et al., 2018).

Previous studies have assessed subjective listening effort under fixed conditions like SNRs (Zekveld et al., 2011; Ohlenforst et al., 2017b, 2018). However, recent research has indicated that listening effort may not correlate monotonically with task demands. Variations in effort follow an inverted U-shaped curve, suggesting that listeners may “give up” when challenged and expend less effort at lower SNRs (Ohlenforst et al., 2017a). Studies showed listeners with normal hearing and impairment hearing may allocate different auditory resources for speech understanding (Zekveld et al., 2011; Ohlenforst et al., 2017a,b).

Knowledge about the effects of NR influences on listening effort for individuals with severe-to-profound hearing loss remains limited. Unlike mild and moderate hearing loss, the abilities of individuals with severe-to-profound hearing loss do not show a clear correlation with the degree of hearing loss. In the case of severe hearing loss, speech recognition in quiet was associated with multiple factors, including the degree of hearing loss, spectral resolution and the presence of dead regions. However, for speech in noise, the variance in performance was only modestly explained by amount of hearing loss (Souza et al., 2018).

The purpose of the present study was to assess aided speech recognition, listening effort, and sound quality ratings in noise conditions with and without NR, implemented within a commercially available hearing aid for adults with severe-to-profound hearing loss.

## Materials and methods

### Participants

The sample size was computed based on the measure of subjective listening effort. Power analysis was performed using R software (version 4.3.1) based on the simulation of the data from five pilot participants. A linear mixed effects model was fitted to the data and the mixed power function from the mixedPower package (Kumle et al., 2021) was applied to estimate power for different sample size. The simulation was based on 1,000 runs with a varying sample size from 18 to 26. To ensure that a smaller significant effect size can also be detected, a new parameter SESOI (smallest effect size of interest) was added to the mixedPower function. The simulation results were based on the dataset as well as the defined estimates at the same time. Results suggested that a sample size of 20 subjects should be sufficient with a power over 0.8.

**Figure 1:**
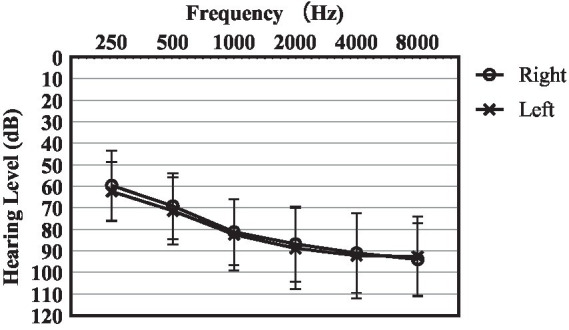
The average air-conduction hearing thresholds of study participants. Error bars show the standard deviations.

Twenty-five Mandarin-speaking adults (9 males and 16 females) aged between 26 and 77 years old (M = 46.5, SD = 18.4) were recruited. All participants were diagnosed with sloping severe-to-profound hearing loss. The average pure-tone audiometric thresholds between 500 and 4,000 Hz for both ears were above 65 dB HL. Figure 1 shows the group average pure-tone thresholds with standard deviation. All participants met the following inclusion criteria: (1) symmetric sloping sensorineural hearing loss (i.e., interaural difference ≤ 15 dB at all octave frequencies from 250 to 8,000 Hz) with air-bone gaps at each frequency ≤ 15 dB; (2) normal middle ear function as indicated by tympanometry and otoscopy examinations; (3) normal cognitive ability screening with MoCA-basic version of Chinses (passing score: 23/30; Chen et al., 2016). (4) At least 1 year hearing aid usage. (5) native Mandarin speakers in daily life. Among the 25 participants, 17 used Phonak brand hearing aids, 4 used Oticon, 2 used Resound, and 2 used Starkey.

### Hearing aids fitting

During the study, each participant wore a pair of Phonak (Stäfa, Switzerland) Naida Paradise 90 UP hearing aids programmed in Phonak Target fitting software (v. 7.1) with Noahlink Wireless. To ensure proper amplification of sound and to limit potential acoustic feedback, custom hard silicone ear molds were made, and the vent size was selected based on the recommendation of the fitting software. The feedback measurement was then performed using the “Real ear and feedback measurement” module in the Phonak Target fitting software to ensure that there was no feedback problem or gain limitation. The APD-Contrast (Adaptive Phonak Digital Contrast) 2.0 fitting prescription was chosen as the target. The initial gain level was set to 100%. Fine-tuning was accomplished by playing a practice list of CMNmatrix (Hu et al., 2018) sentences at 65 dBA SPL from a speaker 1 meter in front of the participant. If the loudness was not appropriate, the broadband frequency gain for the 65 dB SPL input level was adjusted until the participant was satisfied. If participants reported an occlusion problem, the “Occlusion Compensation” feature was turned on and adjusted according to their feedback. To avoid touching by mistake, the button function for volume adjustment was disabled. For tests with speech in noise (SpiN) program, three manual programs based on SpiN were created. These three manual programs differ only in NR strength, namely off, moderate (12) and strong (20) accordingly. For tests with speech in loud noise (SpiLN) program, the same three manual programs were created similarly. These two different base programs were tested in separate appointments. Except from NR setting, the other advanced features such as noise reduction, directionality, etc. were kept the same within each base program.

### The NR function

**Figure 2:**
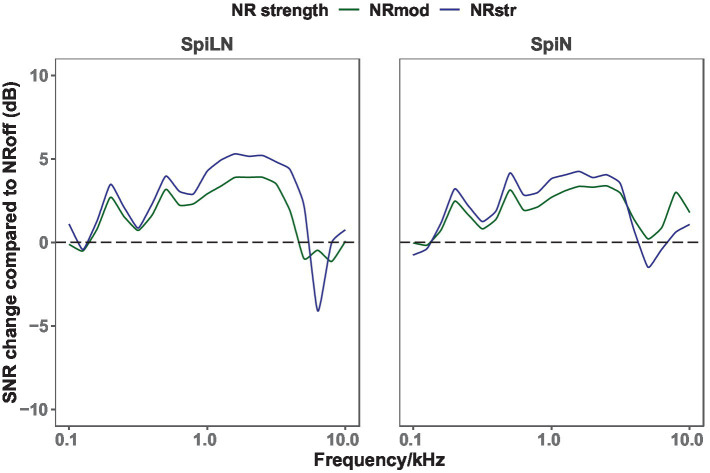
Increased SNR over frequency range of 0.1–10 kHz for NR_mod_ and NR_str_ compared to NR_off_. The measurement was performed using Hagerman and Olofsson (2004) phase invert method. The test setup was same as speech recognition test in Figure 3.

The NR algorithm used in this study (Dynamic Noise Cancelation, DNC) is a spatial noise reduction feature that works in combination with a directional beamformer to improve the signal-to-noise ratio in challenging situations. It is microphone dependent and is only activated when the beamformer is fully activated and switches off as soon as the SNR reaches 18 dB and higher. NR can be adjusted from off to weak, moderate and strong levels in Phonak Target and in myPhonak APP. In Target, the NR appears on a 20-point slider: 0 is off, 1–9 is weak, 10–16 is moderate and 17–20 is strong. A stronger NR setting leads to higher attenuation of noise, which in turn provides a higher SNR benefit (Figure 2).

### Perceptual tasks and outcome measures

#### Speech recognition in noise

The speech reception thresholds (SRTs) were measured adaptively by open-set Mandarin Chinese matrix (CMNmatrix) Sentence Test (Hu et al., 2018). The CMNmatrix sentence contains five words (name-verb-numeral-adjective-noun). There are 10 possible alternatives for each word category, which are randomly combined into syntactically fixed, grammatically correct but semantically unpredictable sentences. During the test, participants responded by repeating what they heard, and the investigator outside of the sound booth marked the correct answers. The SRT was defined as the signal-to-noise ratio at which a listener yielded 50% speech intelligibility.

**Figure 3:**
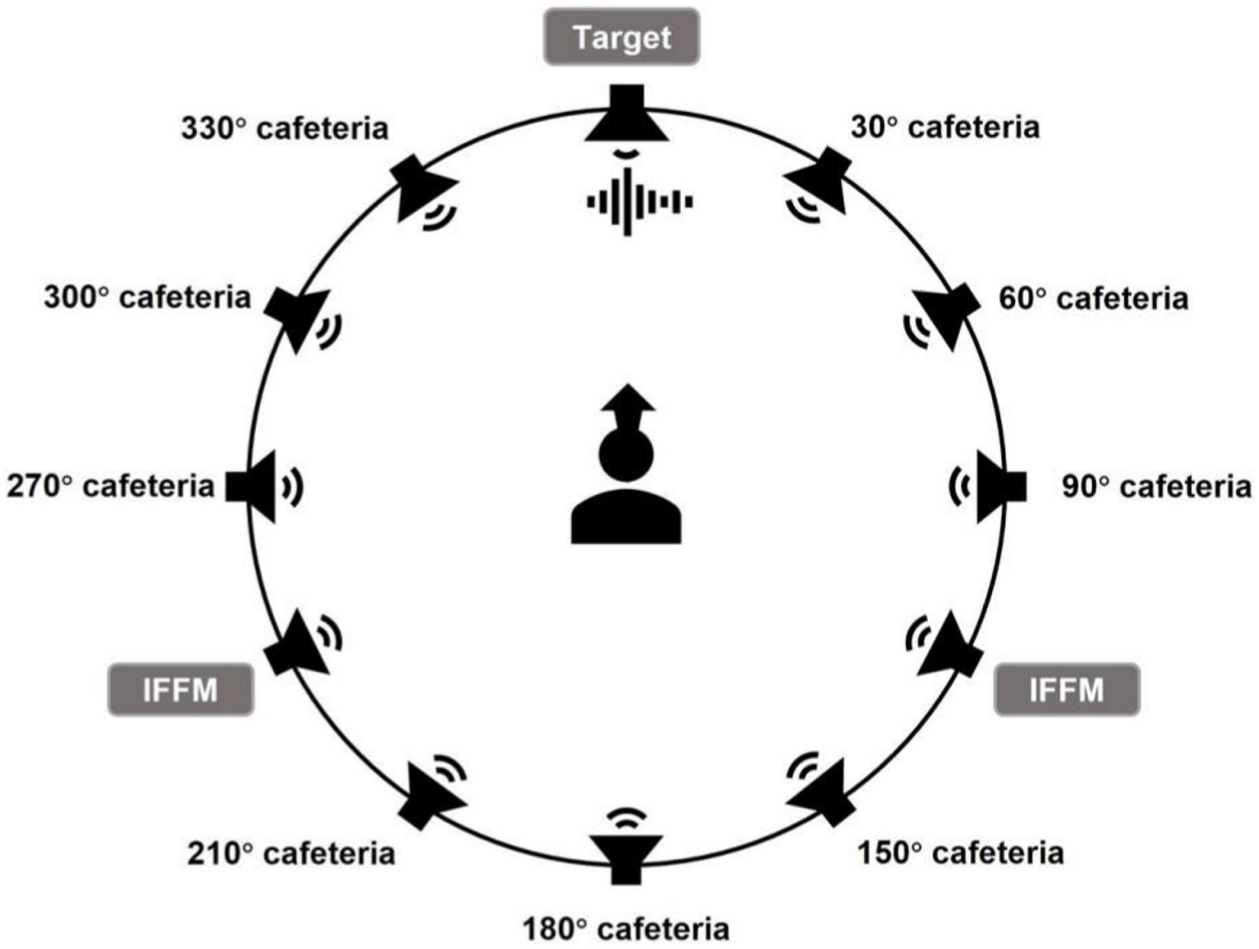
Diagram of speaker and stimuli configuration for ACALES and CMNmatrix sentence test; the radius of speaker circle is 1.4 m.

Test setup of the sound field is shown in Figure 3. The target sentences were presented from 0° azimuth in front of the participant with competing noise from the remaining 11 speakers. Noise sources consisted of two parts. International Female Fluctuating Masker (IFFM) stimuli were used for speakers at 120° and 240° azimuth. Cafeteria noise (recorded in a restaurant during its peak hours with ZOOM H6 in Shanghai) that has been filtered to have the same long-term average spectrum as the speech signal were emitted from the remaining nine speakers. This was considered an ecologically valid noisy situation, which incorporated both stationary and modulated noise source. The level of each cafeteria noise source was approximately 56 dB A, resulting a total cafeteria noise level of 65 dB A. The level of each IFFM source was approximately 63 dB A, resulting a total IFFM level of 65 dB A. In this case, the overall competing noise level was set to 68 dB A. Against the fixed noise level, the sentence level was automatically adjusted according to the Oldenburg sentence test (OLSA) procedure (Brand and Kollmeier, 2002). The initial SNR level was 5 dB, i.e., the sentence level began at 73 dB A, and then was adaptively changed according to the correctness of the participants’ responses.

Before the test, participants were required to review all the test words printed out on a paper to ensure they were familiar with these words especially the names. Two practice lists were administered with NR_off_ to ensure that the participants fully understood the test procedure and to reduce the training effect. In the formal session, one sentence list was tested for each NR strength condition and the order of testing was randomized for three NR strengths.

#### Subjective listening effort

**Figure 4:**
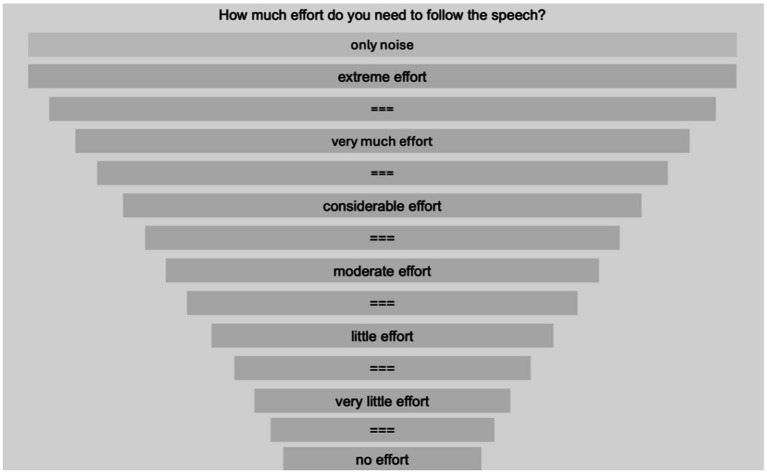
Response interface of ACALES test. See Appendix 1 for the Mandarin text.

The listening effort test was administered using Adaptive Categorical Listening Effort Scaling (ACALES; Krueger et al., 2017) method. The test was performed using MATLAB software (MathWorks, Natick, MA) and was configured with female CMNmatrix sentences being played from 0° azimuth speaker and noise sources present from the remaining 11 speakers. The competing noise for ACALES was the same as that of the CMNmatrix sentence test (Figure 3). The level of the noise was held constant at 68 dBA and the level of the target speech was changed adaptively in order to achieve desired SNRs. For each trial, a group of three sentences was played. Participants were required to rate how much effort they need to follow the speaker at the current SNR by choosing from the 14 categories ranging from “effortless” to “extremely effortful” with one additional “only noise” option. If no response was made after 5 s, another group of sentences were played at the same SNR. For more details about the test structure please see Krueger et al. (2017). Participants responded by mouse clicking with a screen placed directly in front of them (see Figure 4 for the interface).

Since the paradigm was less intuitive as the speech recognition test, adequate training was provided before the formal test. The practice consisted of a maximum of 20 trials presented at SNRs in the range from −25 to 30 dB. The custom MATLAB program presented the training results by plotting the selected listening effort category as function of SNRs in a scatterplot. If the dots were distributed randomly and not even a weak trend of increasing effort with decreasing SNRs was observed visually, the participant received re-instruction and a second practice test. NR settings for the practice test was randomly selected for each participant. For the formal test, the order of three NR strength settings was also randomized across participants.

#### Sound quality assessment

**Figure 5:**
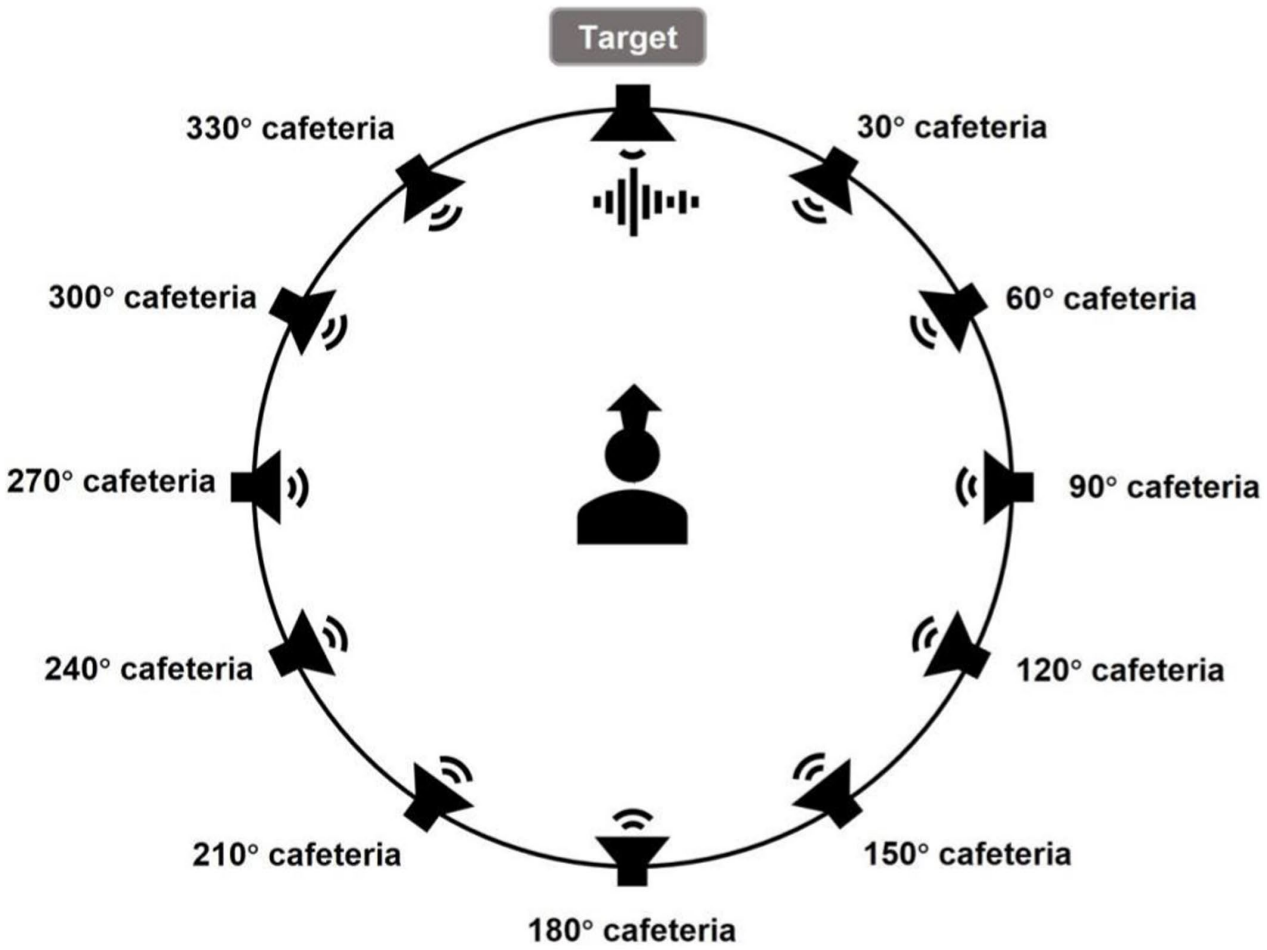
Diagram of speaker and stimuli configuration for subjective sound quality assessment; the radius of the speaker circle is 1.4 m.

**Figure 6:**
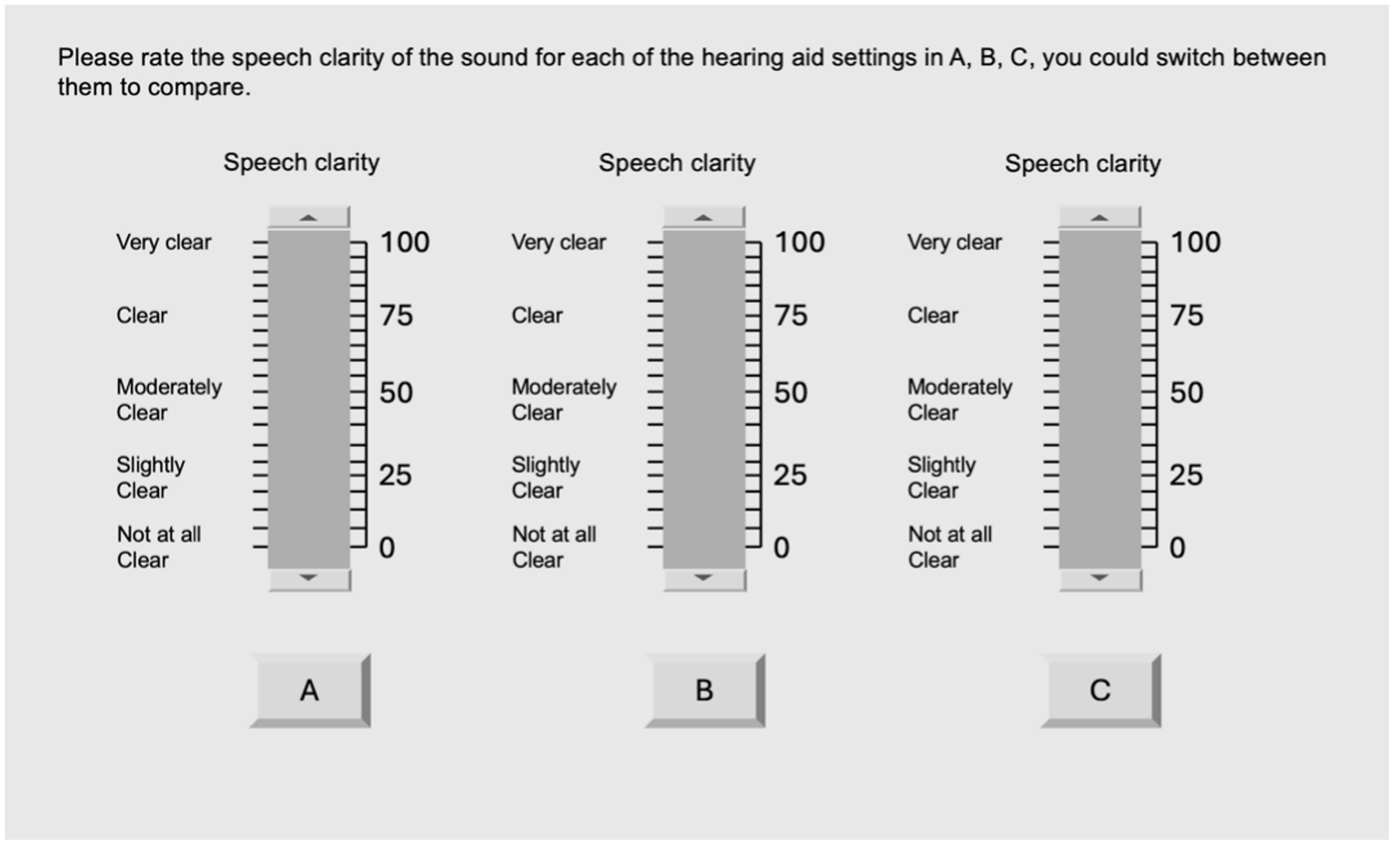
Participant’s screen for the attribute of speech clarity as an example. See Appendix 2 for the Mandarin text.

Sound quality was assessed by comparing the processing effect of three manual programs that differ only in NR strength in real time. A custom MATLAB program that can communicate with the fitting software Target was developed, in which three manual programs were represented by three buttons. When one button was clicked, the underlying HA program was activated by the fitting software. The test sound scene included a target speech presented from the front loudspeaker at 0° azimuth and cafeteria noise presented simultaneously from the remaining 11 loudspeaker (Figure 5). The speech signal at 66 dB A was a 15-s excerpt from a Chinese passage (The North Wind and the Sun) recorded by a female speaker. The combined level of 11 noise sources was 66 dB A, resulting in an SNR of 0 dB. Participants were seated at the center of the speaker circle with an LCD monitor placed in front of them. They were asked to rate the sound quality of the speech signal in dimensions of listening comfort, noise suppression, speech clarity, and overall satisfaction. For each attribute, there were three sliders that the participant can give the rating score for each of the three programs (see Figure 6 for the interface). The range of the slider was from 0 to 100 with five categorical labels at each 25 intervals. The categorical labels and corresponding number of ticks for each attribute are shown in Table 1. The participant was able to switch between three programs with different NR strengths (hidden by “A,” “B,” “C”) by clicking on the buttons at the bottom of the screen (Figure 6). At the top of the screen, the title of the test was shown to instruct participants which attribute to rate at the current trial. Before the formal test, a training session was provided to familiarize the participants with the method and the rating scale. In the formal test, both test and retest were included for each attribute.

**Table 1:** Rating range and categorical labels for sound quality rating.

Listening comfort	Noise suppression	Speech clarity	Overall impression
0-Not at all comfortable	0-No suppression	0-Not at all clear	0-Not at all satisfied
25-Slightly comfortable	25-Little suppression	25-Slightly clear	25-Slightly satisfied
50-Moderately comfortable	50 -Moderate suppression	50-Moderately clear	50-Moderately satisfied
75 -Comfortable	75-Much suppression	75-Clear	75-Satisfied
100-Very comfortable	100-Strong suppression	100-Very clear	100-Very satisfied

### Procedures

All participants were tested at three separate sessions to complete all the measures within a 2-week period. The interval between each visit was 3 to 4 days. Tests were conducted in a sound booth with background noise below 30 dB A in Beijing Tongren Hospital. Twelve speakers (Audioengine 2^+^) were arranged in a circle with a radius of 1.4 m. The touchscreen monitor (Philips) used in listening effort and sound quality rating to present response interface was 50 cm long and 30 cm wide.

In the first session, after obtaining the informed consent from participants, otoscopy, pure tone audiometry (both air conduction and bone conduction) and tympanometry were implemented to check hearing condition, followed by MoCA screening. Individual ear impression was taken at last. In the second visit, speech recognition in noise test, subjective listening effort rating and sound quality assessment were conducted for three manual programs of different NR strengths (NR_off_, NR_mod_, NR_str_) with SpiN as the base program. In the third visit, all the tests and settings were kept the same as those in the second visit, except that SpiLN was used as the base program to create manual programs. In these two sessions, the order of three outcome measures was fixed, but the order of NR strength conditions was randomized within each measure. Break was scheduled between tests or whenever required by the participants.

### Statistical analysis

The above three outcomes were measured repeatedly with three NR strengths under each of the two HA programs (SpiN and SpiLN). For all three measures, data of SpiN and SpiLN was analyzed separately. The individual rated listening effort was fitted as a two-slope function of the SNR using the BX fitting method (Oetting et al., 2014) which was recommended by Krueger et al. (2017). The data points of the rating category “only noise” were excluded. The SNR values at “moderate effort” (7 ESCU-Effort Scaling Category Units) for each participant and condition were extracted. A repeated measures analysis of variance (rmANOVA) with SNRs at “moderate effort” as the dependent variable and NR strength condition as the independent variable was performed to see if SNRs required to reach “medium effort” were different between NR strength conditions. Data of speech recognition in noise test was analyzed by the repeated measures one-way ANOVA with SRT50 as the dependent variable and NR strength conditions as the independent variable. For sound quality assessment, rating scores were averaged across the test and retest for analysis. Repeated measures one-way ANOVA with rating scores as the dependent variable and NR strength conditions as the independent variable was performed for each of the four attributes. Friedman’s test was used instead if the normal distribution assumption was not met. Statistical analyses were conducted using R software (version 4.3.2; R Core Team, 2021) with “bruceR” package (Bao, 2023) for ANOVA and “ggplot2” package (Wickham, 2016) for graphs.

## Results

### Speech intelligibility

**Figure 7:**
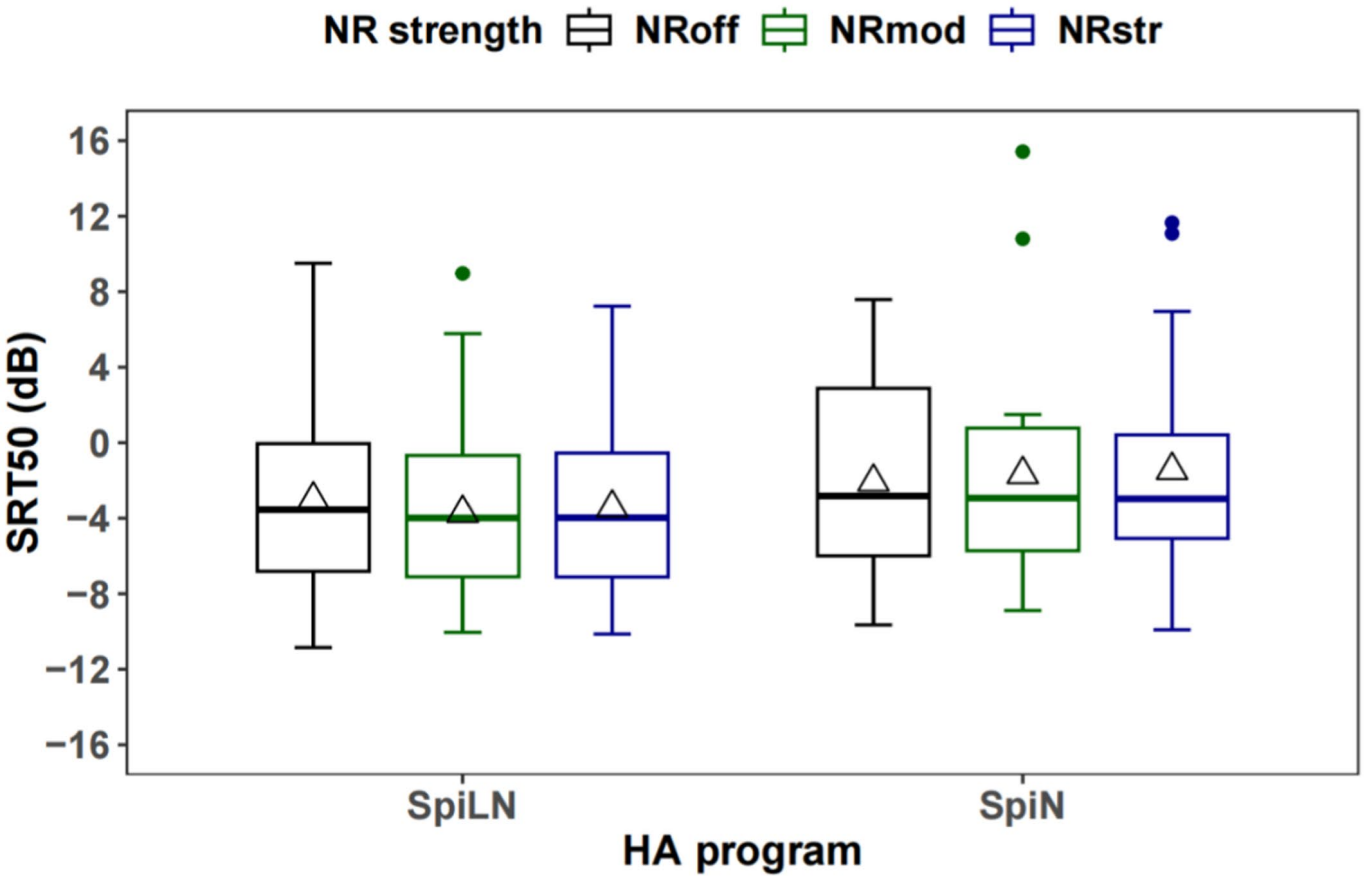
Boxplots of SRTs of three NR strength levels for both SpiLN and SpiN programs. The triangle sign represents the mean. The horizontal line within each box indicates the median. Lines extending from the top and bottom of each box indicate the highest and lowest values within 1.5 times the interquartile range. NR, Noise Reduction; SpiN, speech in noise program; SpiLN, speech in loud noise program.

Figure 7 shows boxplots of SRTs of three NR strength levels for the SpiLN and for SpiN programs. The mean SRTs of −3.0, −3.7, and −3.5 dB were obtained in the three NR conditions for the SpiLN program, respectively. Great variability in results was observed, with SDs of 5.5 dB, 5.1 dB, and 4.9 dB, respectively, in the three NR conditions. The median SRTs of −2.8, −2.9, and −3.0 dB, obtained in the three NR conditions for condition of SpiN, respectively. SRT50s were compared between three NR levels (NR_off_, NR_mod_, NR_str_) in two programs (SpiN or SpiLN) separately. SRTs in each test condition of SpiLN program were normally distributed as verified by the Shapiro–Wilk test. There is no significant effect of noise reduction (NR), *F*(2, 42) = 1.133, *p* = 0.332. For SpiN program, SRTs in NR_mod_ were not normally distributed. Therefore, a non-parametric test Friedman Test was applied, which also indicated no significant effect of NR, *χ^2^*(2) = 3.7, *df* = 2, *p* = 0.157.

### Listening effort

**Figure 8:**
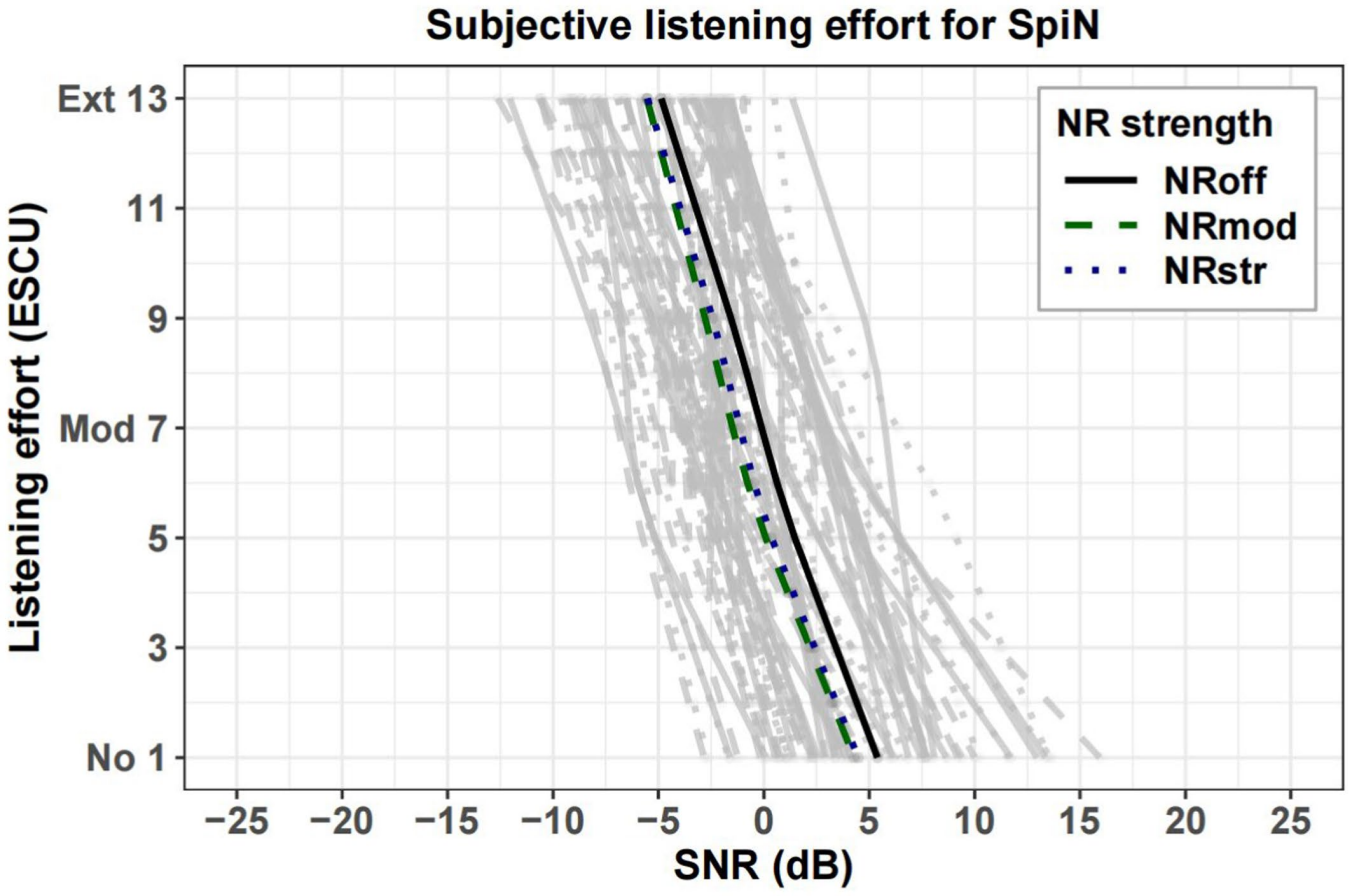
Individual estimated two slope functions for listening effort of each participant (gray lines) and mean estimated two slope functions (highlighted) for NR_off_ (solid line), NR_mod_ (dashed line), and NR_str_ (dotted line) in SpiN program. The rating category “no effort” corresponds to 1 ESCU, “medium effort” to 7 ESCU, and “extreme effort” to 13 ESCU. NR, noise reduction; ESCU, Effort Scaling Category Units.

**Figure 9:**
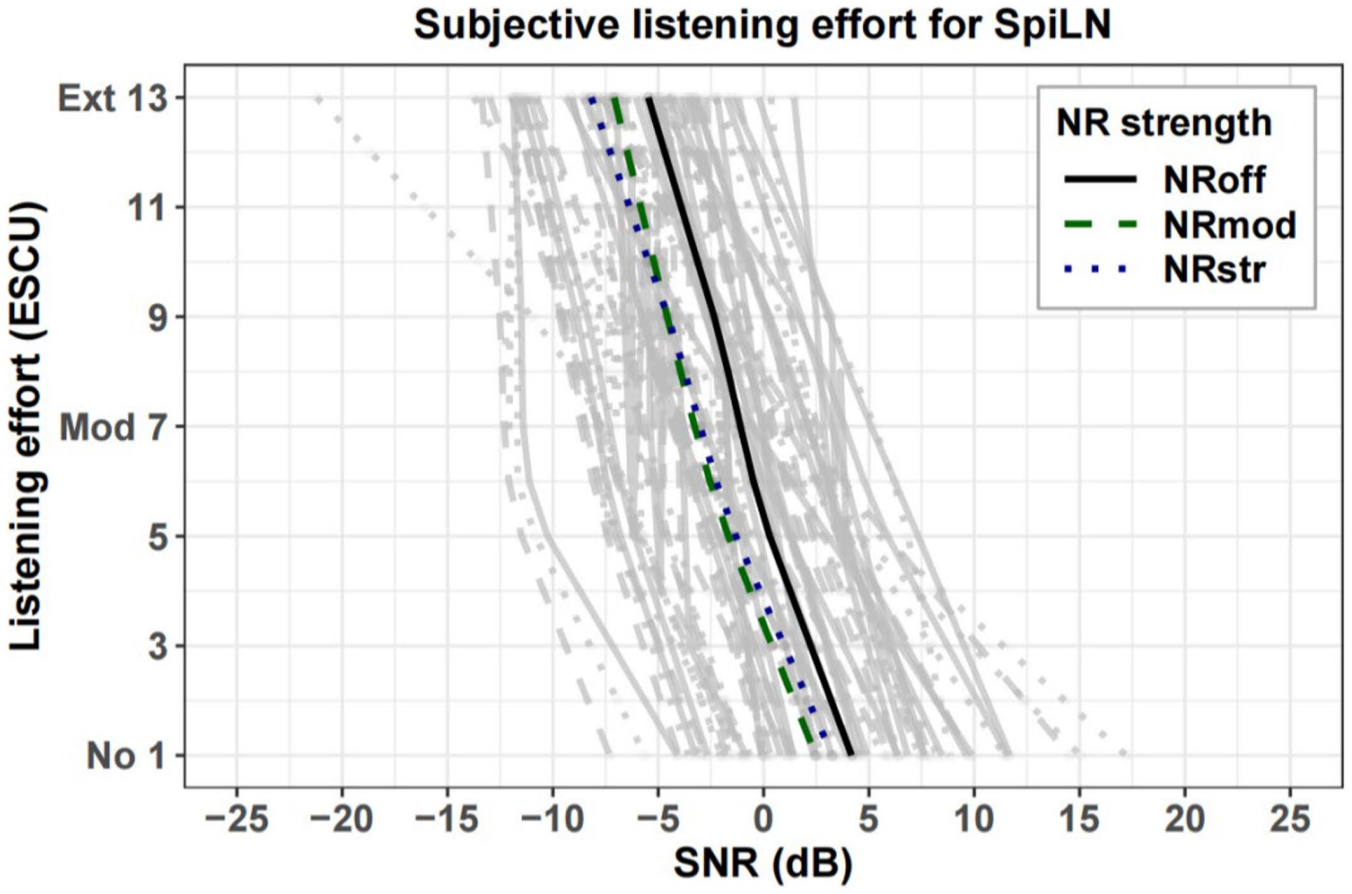
Individual estimated two slope functions for listening effort of each participant (gray lines) and mean estimated two slope functions (highlighted) for NR_off_ (solid line), NR_mod_ (dashed line), and NR_str_ (dotted line) in SpiLN program. The rating category “no effort” corresponds to 1 ESCU, “medium effort” to 7 ESCU, and “extreme effort” to 13 ESCU. NR, noise reduction; ESCU, Effort Scaling Category Units.

**Figure 10:**
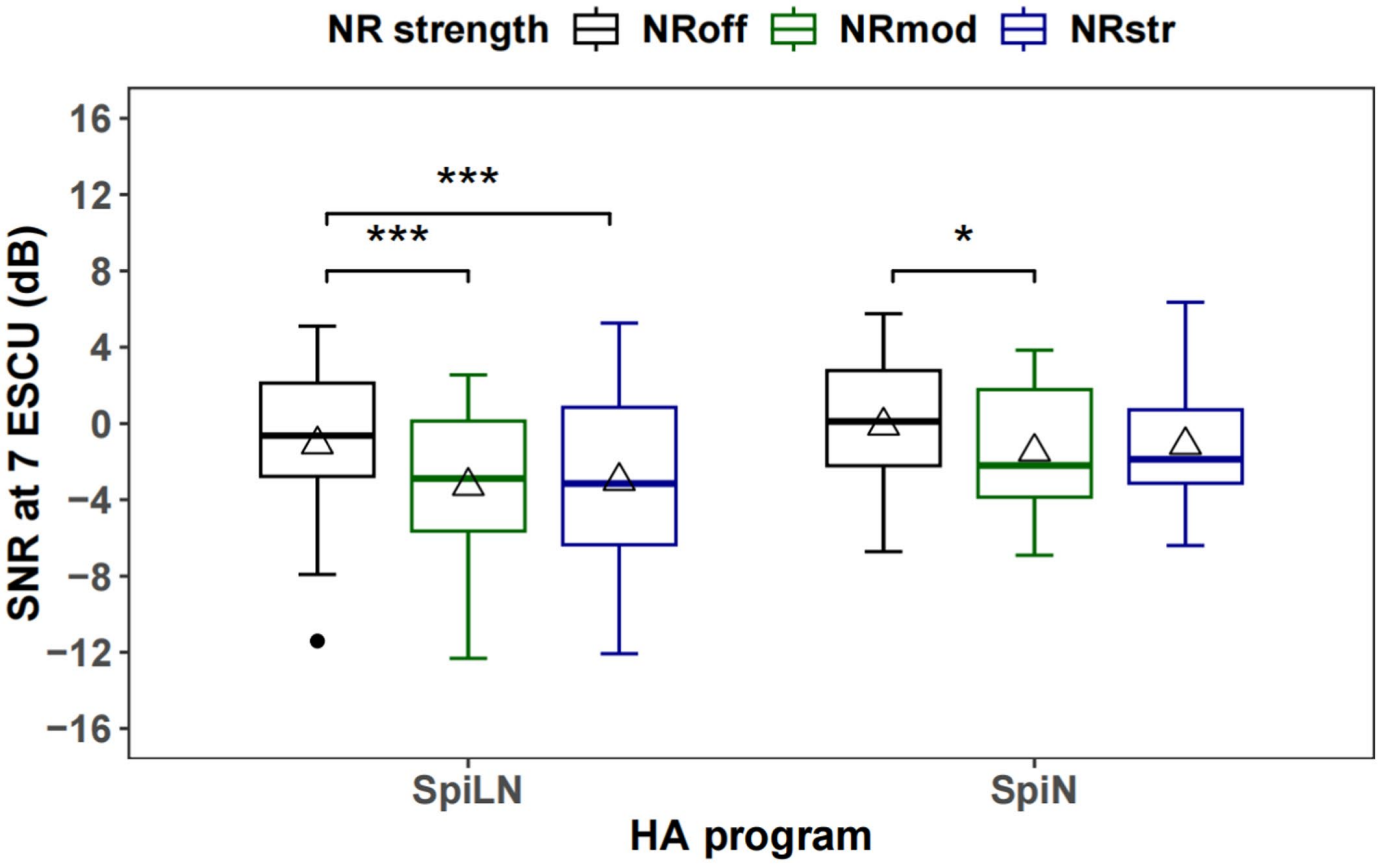
Boxplots of SNR at 7 ESCU, which represents the medium effort rating point in the listening effort function in dB SNR, are presented. The triangle marker indicates the mean, and the horizontal line shows the median. The width of the box depicts the interquartile range, and lines extending from the box represent values within 1.5 times the interquartile range. ^***^p < 0.001, ^*^p < 0.05.

Figures 8, 9 show estimated two-slope functions of listening effort for each individual and the group mean of the results for three NR strengths within the SpiN program an SpiLN program, respectively. The mean listening effort function was calculated by averaging the SNRs for each rating category over all participants. In both HA programs, there is a large amount of variation in the participant’s individual listening effort function. For the group mean function, the perceived listening effort decreased with increasing SNR. Across the entire range of listening effort scaling, the NR_off_ required higher SNR values to achieve similar effort ratings compared to the NR activate conditions (NR_mod_ and NR_str_). Such advantages of NR in SpiN program appeared to be more pronounced roughly between “little effort” (5 ESCU) to “medium effort” (7 ESCU), but more discrete for “no effort” (1 ESCU) and “extreme effort” (13 ESCU). As for SpiLN program, NR activated conditions (NR_mod_ and NR_str_) provided more advantages roughly from “medium effort” (7 ESCU) to “considerable effort” (9 ESCU). In terms of quantitative analysis, in Figure 10, the SNR values at 7 ESCU were extracted because it was the midpoint of the smooth area of the two-slope listening effort function. For SpiN program, the mean SNR at 7 ESCU was 0 dB for the NR_off_, −1.3 dB for the NR_mod_, and −1.1 dB for the NR_str_ with standard deviations of 3.3, 3.1, and 3.3 dB, respectively. There is a significant effect of noise reduction (NR) settings. Specifically, the difference from NR_off_ was significant for NR_mod_ [*t*(22) = −3.032, *p* = 0.018] but not for NR_str_ [*t*(22) = −2.23, *p* = 0.11]. No significant difference was found between NR_mod_ and NR_str_ [*t*(22) = 0.36, *p* = 1]. In the SpiLN program, the mean SNR at 7 ESCU was −1.1 dB for NR_off_, −3.3 dB for NR_mod_, and −3 dB for NR_str_, with standard deviations of 4.0 dB, 3.8 dB, and 4.2 dB, respectively. Significant differences from NR_off_ were observed with both NR_mod_ [*t*(24) = −6.17, *p* < 0.001] and NR_str_ [*t*(24) = −6.08, *p* < 0.001]. No significant difference was found between NR_mod_ and NR_str_ [*t*(24) = 1.03, *p* = 0.944].

### Sound quality assessment

**Figure 11:**
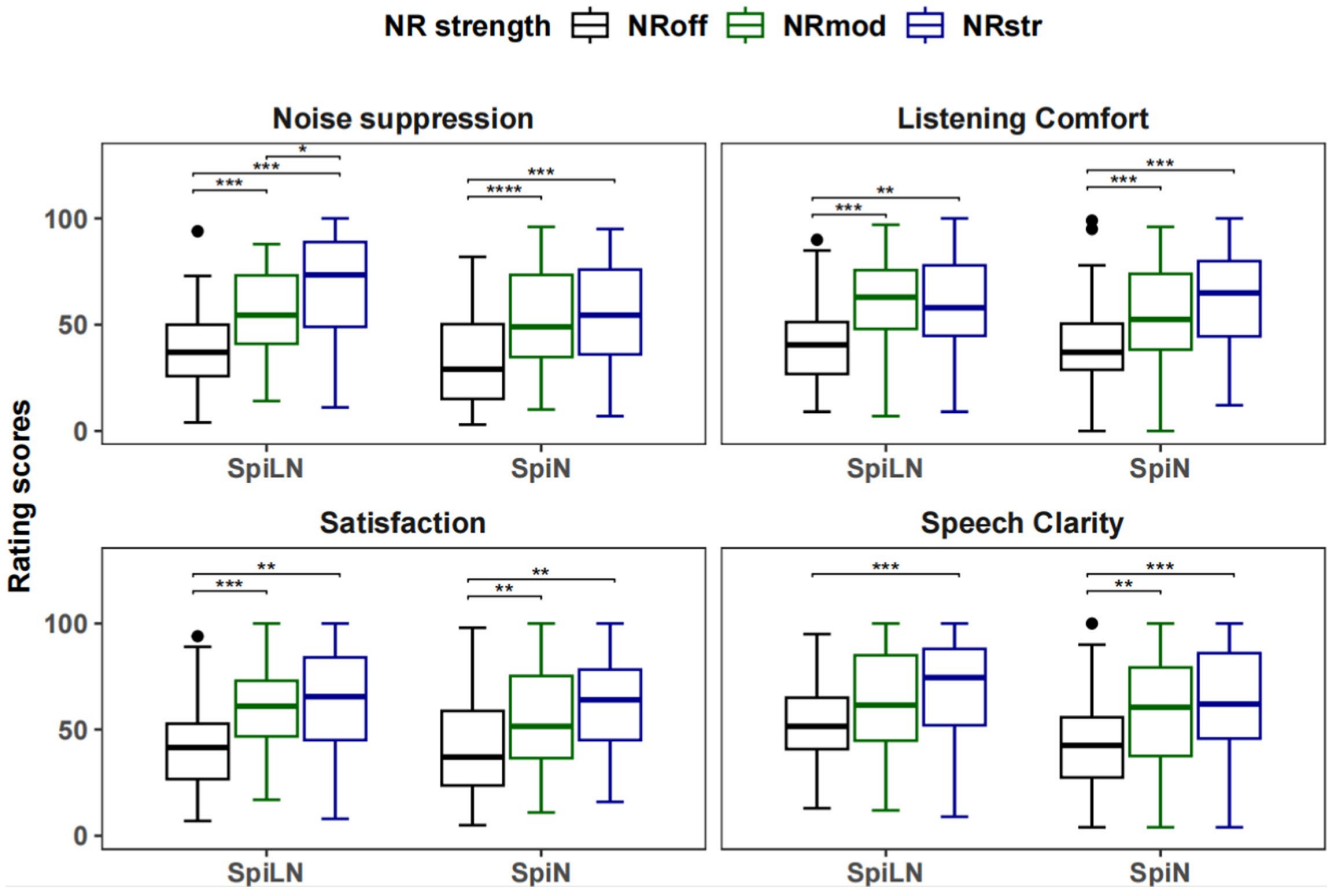
Boxplots of sound quality ratings for four attributes. The median is shown by the horizontal line at the center of each box. The width of the box represents the interquartile range, while lines extending from the top and bottom edges of the box indicate the highest and lowest values within 1.5 times the interquartile range. ^****^p < 0.0001, ^***^p < 0.001, ^**^p < 0.01, ^*^p < 0.05.

Figure 11 plots the average sound quality ratings from the 24 participants in the three NR strengths. One participant’s data was missing. For the rating of noise suppression in the SpiLN program, the analysis yielded significant main effect of NR [*F*(1.461, 33.594) = 18.43, *p* < 0.001]. The participants rated noise suppression significantly higher with NR_str_ than with NR_off_ [*t*(23) = 4.78, *p* < 0.001] and with NR_mod_ [*t*(23) = 2.95, *p* < 0.05]; NR_mod_ was rated significantly higher than NR_off_ [*t*(23) = 4.39, *p* < 0.001]. For SpiN program, the main effect of NR was significant [*F*(1.344, 30.908) = 18.57, *p* < 0.001]. The participants rated noise suppression significantly higher with NR_str_ [*t*(23) = 4.45, *p* < 0.001] and NR_mod_ [*t*(23) = 5.19, *p* < 0.001] than with NR_off_.

In terms of listening comfort and satisfaction ratings, the main effect of NR was significant for both the SpiLN program [Listening comfort: *F*(2, 46) = 11.35, *p* < 0.001; Satisfaction: *F*(1.308, 30.094) = 12.87, *p* < 0.001] and the SpiN program [Listening comfort: *F*(2, 46) = 16.56, *p* < 0.001; Satisfaction: *χ^2^*(2) = 17.58, *p* < 0.001]. For SpiLN program, participants rated significantly higher listening comfort and satisfaction with NR_str_ [Listening comfort: *t*(23) = 3.35, *p* < 0.01; Satisfaction: *t*(23) = 3.45, *p* < 0.01] and with NR_mod_ [Listening comfort: *t*(23) = 4.38, *p* < 0.001; Satisfaction: *t*(23) = 4.80, *p* < 0.001] than with NR_off_. For SpiN program, both sound attributes were rated significantly higher with NR_str_ [Listening comfort: *t*(23) = 4.81, *p* < 0.001; Satisfaction: *Z* = 37, *p* < 0.01] and NR_mod_ [Listening comfort: *t*(23) = 4.41, *p* < 0.001; Satisfaction: *Z* = 50, *p* < 0.01] than with NR_off._

For clarity ratings, the analysis revealed a significant effect of NR for both the SpiLN program [*F*(2, 46) = 9.09, *p* < 0.001] and the SpiN program [*F*(2, 46) = 15.36, *p* < 0.001]. Participants rated significantly higher speech clarity with NR_str_ than with NR_off_ for both the SpiLN program [*t*(23) = 4.26, *p* < 0.001] and the SpiN program [t(23) = 4.68, *p* < 0.001]. The speech clarity was rated significantly higher with NR_mod_ than with NR_off_ only in the SpiN program [*t*(23) = 3.84, *p* < 0.01].

## Discussion

The primary objective of this study was to examine the perceptual outcome of individuals with severe-to-profound hearing loss in response to noise reduction technology. Our assessments encompassed domains related to speech recognition, listening effort, and subjective sound quality. Our research findings indicate that individuals with severe-to-profound hearing loss derived specific benefits from the implementation of noise reduction technologies. These benefits included reduced listening effort and improved subjective sound quality, as observed within the methodological framework of our study. It is important to note, however, that these advantages did not extend to improvements in speech intelligibility.

### Speech intelligibility

No significant difference in speech intelligibility was observed between HA settings with and without NR. While the NR algorithm utilized in this study did not lead to improved speech recognition, it is noteworthy that it did not negatively affect performance, which has also been observed with certain other NR algorithms (Ricketts and Hornsby, 2005). This is consistent with our hypothesis that speech recognition in noise may not be improved by NR processing, but neither is degraded. Previous research has suggested that speech degradation caused by high levels of noise or distorted speech cues could lead to delays in lexical processing (Ben-David et al., 2011; McMurray et al., 2017). On the one hand, the application of NR processing could attenuate a certain amount of background noise, but on the other hand it would introduce some distortion of speech cues (Kates, 2008). If the level of speech cue distortion is high enough to counteract the effect of noise reduction, listeners may not benefit from NR due to the increased cognitive workload in speech processing (Winn et al., 2015; Ward et al., 2017).

For individuals with mild to moderate hearing loss, several studies have reported improvements in speech intelligibility with NR algorithms (Peeters et al., 2009; Ohlenforst et al., 2018; Wong et al., 2018). Ohlenforst et al. (2018) found that sentence recognition performance was significantly better when NR was enabled than when it was not. The study by Wong et al. (2018) revealed that when NR was activated, listeners demonstrated improved speech perception abilities and reported reduced noise annoyance, resulting in improved speech clarity. These findings have been attributed in part to predictable relationships between degree of impairment and auditory-cognitive abilities in individuals with mild to moderate hearing loss (Humes, 2007; Souza et al., 2007). These associations diminished for listeners with more severe hearing loss, whose abilities became more variable and unpredictable.

In this study, NR settings on the hearing aids, being new settings for the participants, might have been perceived as unfamiliar input. This unfamiliarity could lead to an increased reliance on working memory, as participants needed to utilize more cognitive resources to resolve the unfamiliar stimuli (Souza et al., 2015; Ronnberg et al., 2019, 2022). The speech tests employed in this study included cafeteria noise and the International Female Fluctuating Masker (IFFM), incorporating both energetic and informational maskers. These maskers pose distinct challenges: energetic maskers distract, while informational maskers impact more significantly as they engage semantic long-term memory (SLTM; Sorqvist and Ronnberg, 2012). In situations involving informational maskers, working memory capacity (WMC) is a crucial predictor of performance. The findings suggest that high dependency on working memory was not mitigated by prolonged use of hearing aids; a decade of hearing aid use does not diminish this reliance, especially with four-talker (4 T) maskers (Ng and Ronnberg, 2020). Additionally, the study’s speech recognition materials were akin to Hagerman matrix sentences. Ronnberg et al. (2016) found that working memory had a stronger correlation with Hagerman matrix sentences than with HINT sentences, which are driven by everyday contexts. This underscores the significant role of working memory in complex listening environments. Hearing aid users with severe-to-profound hearing loss often exhibited constraints in working memory capacity, which might impair their capability to process and decode speech signals following NR processing. Although NR technology effectively suppressed background noise, this suppression might not adequately compensate for the challenges encountered by these individuals in processing speech information and memory tasks. Consequently, focusing on traditional speech recognition tests might not fully capture the overall improvements in communication achieved through NR technology. Additionally, these tests might lack sensitivity to the enhancements in NR’s top-down processing. Therefore, it was essential to supplement these assessments with subjective evaluations and measures of listening effort to comprehensively evaluate the influence of NR.

### Listening effort

This study investigated the effect of NR schemes on listening effort as indicated by a subjective method in a group of people with sever-to-profound hearing impairment. Our results suggested that the NR processing reduced the listening effort required in noisy conditions for individuals with severe-to-profound hearing loss. Participants preferred either NR_mod_ or NR_str_ over NR_off_, were consistent with previous work demonstrating a reduction in listening effort but no improvement in speech intelligibility when enabling NR (Desjardins and Doherty, 2014; Ohlenforst et al., 2018). In addition, several other studies had shown that participants experienced reduced listening effort when using NR in noisy environments (Sarampalis et al., 2009; Brons et al., 2014; Wong et al., 2018). Despite variations in specific details, overall, these findings confirmed that listening effort evaluations could sensitively capture listeners’ perceived effort.

Kestens et al. (2021) undertook a systematic review to investigate how intersubject differences in cognition could influence the aided benefit for speech understanding and listening effort with bilateral digital hearing aids. The research results demonstrated that the effects of noise reduction technology varied among hearing aid users with different cognitive abilities. For instance, the study conducted by Desjardins and Doherty (2014) did not find a significant relationship between working memory and listening effort when using noise reduction technology. In contrast, Wendt et al. (2017) found that users with better working memory capacities experienced less objectively measured listening effort compared to HA users with less memory capacity when utilizing the noise reduction feature. Furthermore, users with slower processing speeds appeared to benefit more from noise reduction technology in terms of listening effort, indicating that they relied more on hearing aids when dealing with complex listening situations (Desjardins and Doherty, 2014). Although noise reduction technology is expected to theoretically help reduce listening effort, the existing research findings remains inconsistent. This variability in outcomes could be due to the different measurement methods used in various studies, the differences in cognitive abilities among participants, and variations in hearing aid technologies.

Researches suggested that NR might not necessarily enhance speech clarity, but rather it could reduce cognitive effort, a benefit that becomes particularly apparent in listening effort (Sarampalis et al., 2009; Desjardins and Doherty, 2014; Wong et al., 2018). In the patient-centered model, testing for listening effort is crucial. The goal of rehabilitation is to reduce the degree of barriers, activity limitations, and participation restrictions for hearing aid users (World Health Organization, 2001). Subjective listening effort seemed to be related to perceived hearing impairment (Alhanbali et al., 2018), and subjective listening effort could have a negative impact on the quality of life (Pichora-Fuller et al., 2016).

### Sound quality assessment

Overall, the activation of NR provided clear advantages compared to its deactivation across several dimensions, including enhanced noise tolerance, improved listening comfort, increased satisfaction, and better perceived speech signal quality. These trends showed a more favorable and comfortable auditory experiences when noise was selectively attenuated and fine-tuned by NR algorithms.

The findings of our study were consistent with observations that a majority of individuals with hearing impairments tended to employ NR features in their hearing aids (Souza et al., 2018). Clearly, with noise reduction activated, speech and noise that had undergone noise reduction processing became more comfortable for listeners due to the reduction in noise levels. Overall, these subjective assessments of NR evaluation demonstrated positive effects in terms of noise acceptance, user preferences, and enhanced listening comfort. Numerous studies have previously indicated that noise reduction had a positive impact on noise perception and preferences. Studies utilizing the Abbreviated Profile of Hearing Aid Benefit (APHAB; Cox and Alexander, 1995) have consistently shown that noise reduction has a notable impact on reducing aversion to noise (Boymans and Dreschler, 2000; Palmer et al., 2006). Brons et al. (2013) demonstrated that activating noise reduction reduced annoyance caused by noise, although it found no impact on speech naturalness through paired comparison testing. Palmer et al. (2006) discovered that when NR was activated, the annoyance ratings of hearing-impaired participants closely resembled those of individuals with normal hearing. These findings support that noise reduction has the potential to enhance listening comfort and reduce noise-related annoyance. In this study, participants reported improved speech clarity when NR was activated. This apparent discrepancy between objective speech intelligibility and subjective speech clarity ratings may stem from the different conditions under which each was tested. Specifically, speech intelligibility was assessed using an adaptive procedure that varied the signal-to-noise ratio (SNR) within a negative range. It is documented that NR algorithms are more effective at positive SNRs than at negative ones, which could influence these outcomes (Fredelake et al., 2012; Smeds et al., 2015). Furthermore, the benefits in terms of listening ease and comfort found in this study did not coincide with any reduction in speech intelligibility.

## Conclusion

This study highlights that that individuals with severe-to-profound hearing loss can still derive significant benefits from noise reduction technology. These benefits are evidenced by reduced listening effort and improved subjective sound quality ratings. Importantly, these advantages were achieved without compromising speech intelligibility. While the efficacy of the specific NR was confirmed in this study, its clinical relevance, particularly its potential to predict real-world benefits, should be further explored in future research. It’s crucial to note that the findings of this study may not be directly applicable to hearing aids featuring different NR characteristics or to diverse acoustic environments not encompassed within this study. Furthermore, it is essential to assess the benefits of NR when combined with other adaptive features, such as beamformer.

## Data availability statement

The raw data supporting the conclusions of this article will be made available by the authors, without undue reservation.

## Ethics statement

The studies involving humans were approved by the Institutional Review Board at the Beijing Tongren Hospital. The studies were conducted in accordance with the local legislation and institutional requirements. The participants provided their written informed consent to participate in this study.

## Author contributions

RD: Conceptualization, Data curation, Formal analysis, Funding acquisition, Investigation, Methodology, Project administration, Supervision, Validation, Writing – original draft, Writing – review & editing. PL: Data curation, Investigation, Resources, Supervision, Validation, Writing – review & editing. XT: Conceptualization, Formal analysis, Methodology, Project administration, Supervision, Validation, Visualization, Writing – original draft, Writing – review & editing. YW: Data curation, Investigation, Supervision, Validation, Writing – review & editing. YC: Data curation, Investigation, Supervision, Writing – review & editing. JZ: Conceptualization, Data curation, Methodology, Validation, Visualization, Writing – original draft, Writing – review & editing. LY: Conceptualization, Methodology, Resources, Writing – review & editing. SZ: Supervision, Validation, Writing – review & editing. JG: Conceptualization, Methodology, Supervision, Validation, Writing – review & editing. SW: Conceptualization, Funding acquisition, Methodology, Project administration, Resources, Supervision, Validation, Writing – review & editing.
