# Supplementary figures and images for: Influences of noise reduction on speech intelligibility, listening effort, and sound quality among adults with severe to profound hearing loss
Source: Front Neurosci. 2024 Jul 23;18:1407775. doi: 10.3389/fnins.2024.1407775 (PMC11301946; doi:10.3389/fnins.2024.1407775)

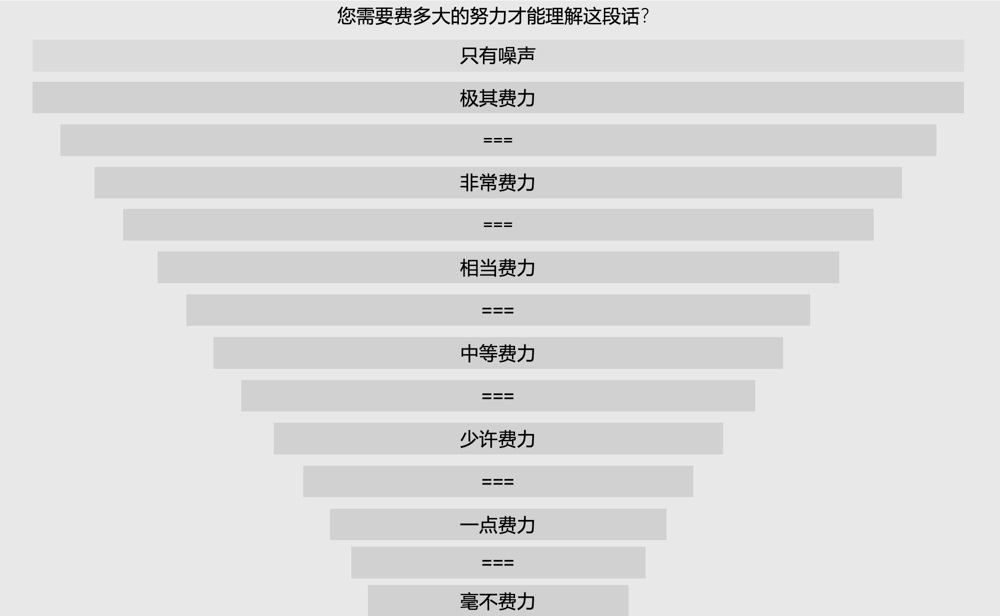

Supplement: Supplementary file 1 [file Image_1.JPEG]

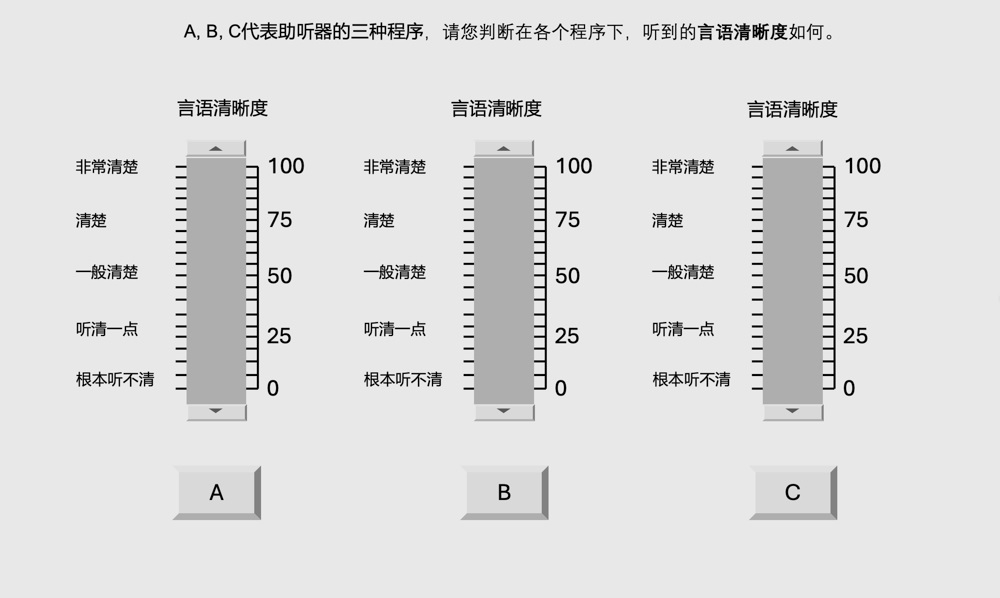

Supplement: Supplementary file 2 [file Image_2.JPEG]
